# Construction and comprehensive analysis of a competitive endogenous RNA network to reveal potential biomarkers for the malignant differentiation of glioma

**DOI:** 10.1097/MD.0000000000027248

**Published:** 2021-10-01

**Authors:** Xin Li, Jingwen Zhang, Min Zhang, Xianghua Qi, Shiyuan Wang, Jing Teng

**Affiliations:** a Weifang Traditional Chinese Medicine Hospital, WeiFang, China; b School of Acupuncture and Massage, Shandong University of Traditional Chinese Medicine, JiNan, China; c School of Statistics, Renmin University of China, Beijing, China; d Affiliated Hospital of Shandong University of Traditional Chinese Medicine, JiNan, China; e School of Traditional Chinese Medicine, Shandong University of Traditional Chinese Medicine, JiNan, China; f First School of Clinical Medicine, Shandong University of Traditional Chinese Medicine, JiNan, China.

**Keywords:** competing endogenous RNA, glioblastoma, glioma, long noncoding RNA, microRNA

## Abstract

**Background:**

Long noncoding RNAs (lncRNAs) can act as microRNA (miRNA) sponges to regulate protein-coding gene expression; therefore, lncRNAs are considered major components of the competitive endogenous RNA (ceRNA) network and have attracted growing attention. This study explored the regulatory mechanisms and functional roles of lncRNAs as ceRNAs in the malignant differentiation of low-grade glioma (LGG) to glioblastoma (GBM) and their potential impact on the prognosis of patients with GBM.

**Methods:**

LncRNA and messenger RNA (mRNA) data were extracted from the Cancer Genome Atlas (TCGA) database from 156 GBM samples and 529 LGG samples. Separately, the miRNA expression data were downloaded from the Gene Expression Omnibus database, with the GSE112009 dataset containing miRNA expression data from 10 GBM samples and 15 LGG samples. Weighted gene coexpression network analysis was performed to screen the glioma grade-related lncRNAs. Then, a ceRNA network was established. The database for annotation, visualization, and integrated discovery was adopted to conduct functional enrichment analysis based on 57 upregulated differentially expressed mRNAs in the ceRNA network. Finally, Kaplan–Meier curves were created for the survival analysis of 13 hub lncRNA by combining the clinical data of GBM patients in TCGA.

**Results:**

A ceRNA network including 16 lncRNAs, 18 miRNAs, and 78 mRNAs specific to the malignant differentiation of LGG to GBM was established. The 57 upregulated differentially expressed mRNAs in the ceRNA network were significantly enriched in 35 gene ontology terms and 5 pathways. The survival analysis showed that 2 lncRNAs (LINC00261 and HOXA10-AS) were prognostic biomarkers for patients with GBM in TCGA.

**Conclusion:**

The proposed ceRNA network may help elucidate the regulatory mechanism by which lncRNAs function as ceRNAs and contribute to the malignant differentiation of LGG to GBM. Importantly, the candidate lncRNAs, miRNAs, and mRNAs involved in the ceRNA network can be further evaluated as potential therapeutic targets and prognostic biomarkers for GBM.

## 1 Introduction

Gliomas are the most common primary malignant brain tumors, accounting for 80% of all malignant primary brain and central nervous system tumors, with an annual age-adjusted incidence of 5.65 per 100,000 people in the United States.^[1]^ In general, gliomas are classified into 4 grades based on histology according to the World Health Organization (WHO): grades I and II are considered low-grade gliomas (LGGs), while grades III and IV are considered high-grade gliomas. Glioblastomas (GBM), classified as WHO grade IV gliomas, compose approximately 60% of all high-grade gliomas and are more common in the fifth through seventh decades of life, with a median overall survival period of about 16 to 18 months.^[2]^ LGGs are far from the benign tumors that they were once thought to be. In fact, LGGs are rarely radiologically stable. Careful imaging analysis revealed their average growth rate of 5 mm/yr^[3,4]^; a growth rate of >8 mm per year is associated with a poor prognosis.^[3]^ Furthermore, they all possess the ability to grow, invade, and inevitably to undergo malignant differentiation. Therefore, it is critically important to identify biomarkers for GBM and to investigate the biological mechanisms of LGG malignant differentiation.

Many studies have confirmed that a complicated posttranscriptional regulatory network allows for long non-coding RNAs (lncRNAs), messenger RNAs (mRNAs), and other RNAs to compete with microRNAs (miRNAs) via acting as natural miRNA sponges by virtue of sharing no <1 miRNA response element.^[5]^ Interactions between competitive endogenous RNAs (ceRNAs) through sharing miRNAs indicate a new pathway of gene regulation, which has key effects in cancer progression.^[6–8]^ The discovery of ceRNAs requires the reassessment of our understanding of gene regulatory networks and raises the possibility of proposing a new molecular mechanism, both of them may be potential targets for gene treatment.^[9–11]^

Recently, a growing number of studies have verified that the lncRNA–miRNA–mRNA regulatory network plays a critical role in the progression and pathogenesis of several conditions, including bladder cancer, gastric cancer, and other malignant tumors.^[12–14]^ Yang et al^[12]^ demonstrated that LINC01133 inhibits gastric cancer progression and metastasis by acting as a ceRNA for miR-106a-3p to regulate APC expression and the Wnt/β-catenin pathway. Miao et al^[13]^ discovered that LNIC00612 modulates the expression of E-cadherin and vimentin by competitively sponging miR-590 to elevate the expression of PHF14, thus affecting bladder cancer cellular epithelial–mesenchymal transition. Finally, Zhou et al^[14]^ confirmed that circRASSF2 modulates papillary thyroid carcinoma progression through the miR-1178/TLR4 pathway. However, there have been few studies conducted on the involvement of ceRNA-mediated mechanisms in the transformation of LGG into GBM.

Here, we divided WHO grade III glioma samples to be LGG samples; investigated differences in RNA expression patterns between total 544 LGG samples (including WHO grades I, II, and III) and 166 GBM samples; and constructed a ceRNA network including 16 lncRNAs, 18 miRNAs, and 78 mRNAs. These candidate genes involved in the ceRNA network may become potential therapeutic targets or diagnostic biomarkers for GBM.

## 2 Materials and methods

### 2.1 Data collection and preprocessing

Available lncRNA-seq and mRNA-seq data from 529 LGG samples (of WHO grades I, II, and III) and 156 GBM samples were collected from the Cancer Genome Atlas (TCGA) database (https://tcga-data.nci.nih.gov/) on February 1, 2020. Log2(x+1) transformation was performed on all gene expression data. We normalized the downloaded data using the trimmed mean of the M value (TMM) normalization method of the “edgeR” package in the R software (version 3.6.2; R Foundation for Statistical Computing, Vienna, Austria).^[15]^ When an RNA had duplicate data, the average RNA expression was used. This study fully met the publication requirements of TCGA. The miRNA expression data were downloaded from the Gene Expression Omnibus (GEO) database (www.ncbi.nlm.nih.gov/geo). The GSE112009 dataset contained miRNA expression data from 10 GBM tissue samples and 15 LGG (including 5 WHO grade I, 5 WHO grade II, and 5 WHO grade III) tissue samples. In this dataset, the data were analyzed with RMA (Affymetrix, Santa Clara, CA) data for normalization. The lncRNAs, miRNAs, and mRNAs with average expression values of >1 were retained and low-abundance RNAs were eliminated. The study followed the guidelines of TCGA and GEO, thus, the approval of an ethics committee was not required.

### 2.2 Identification of differentially expressed genes

Differentially expressed mRNAs (DEmRNAs), miRNAs (DEmiRNAs), and lncRNAs (DElncRNAs) in LGG and GBM samples were detected by “edgeR” in the R software.^[15]^ Statistical significance was defined as |log2-fold change (FC)| > 1 with *P*-value <.05. Based on the annotation of the Ensembl database (http://www.ensembl.org/index.html), DElncRNAs and DEmRNAs were defined and encoded. Annotation information of miRNAs was obtained using the Affymetrix Multispecies miRNA-4 Array. Volcano plots of DERNAs were plotted using the R package “ggplot2.”

### 2.3 Weighted correlation network analysis of lncRNAs

Weighted gene coexpression network analysis (WGCNA) was performed to screen glioma grade-related lncRNAs. In this coexpression network, the expression data of 9711 lncRNAs from 529 LGG samples and 156 GBM samples after Log2(x+1) transformation and elimination of low-abundance RNAs were used to construct a weighted gene c-expression network through the WGCNA package of the R software.^[16]^ Gene correlation is described as a network in which the relationship between the connected genes is represented by the weight. Each gene is described as a node. The edge weight between the connected nodes is the pairwise Pearson coefficient. During gene correlation network construction, an adjacency matrix and an adjacency function were defined. Using the adjacency function, the coexpression similarity between genes can be described as the connection strength. The node dissimilarity is input to hierarchical clustering to define network modules. From the clustering tree, many gene coexpression modules can be discovered. In the construction of the hierarchical clustering tree, a dynamic shear algorithm based on tree branch shape is adopted.^[17]^ The relation between modules and glioma grade was analyzed by calculating the Pearson correlation coefficient, and then screening the glioma grade-related lncRNAs. Glioma grade-related DElncRNAs which were the intersection lncRNAs of glioma grade-related lncRNAs and DElncRNAs, used to construct the ceRNA network.

### 2.4 Construction of a ceRNA network

**Figure 1:**
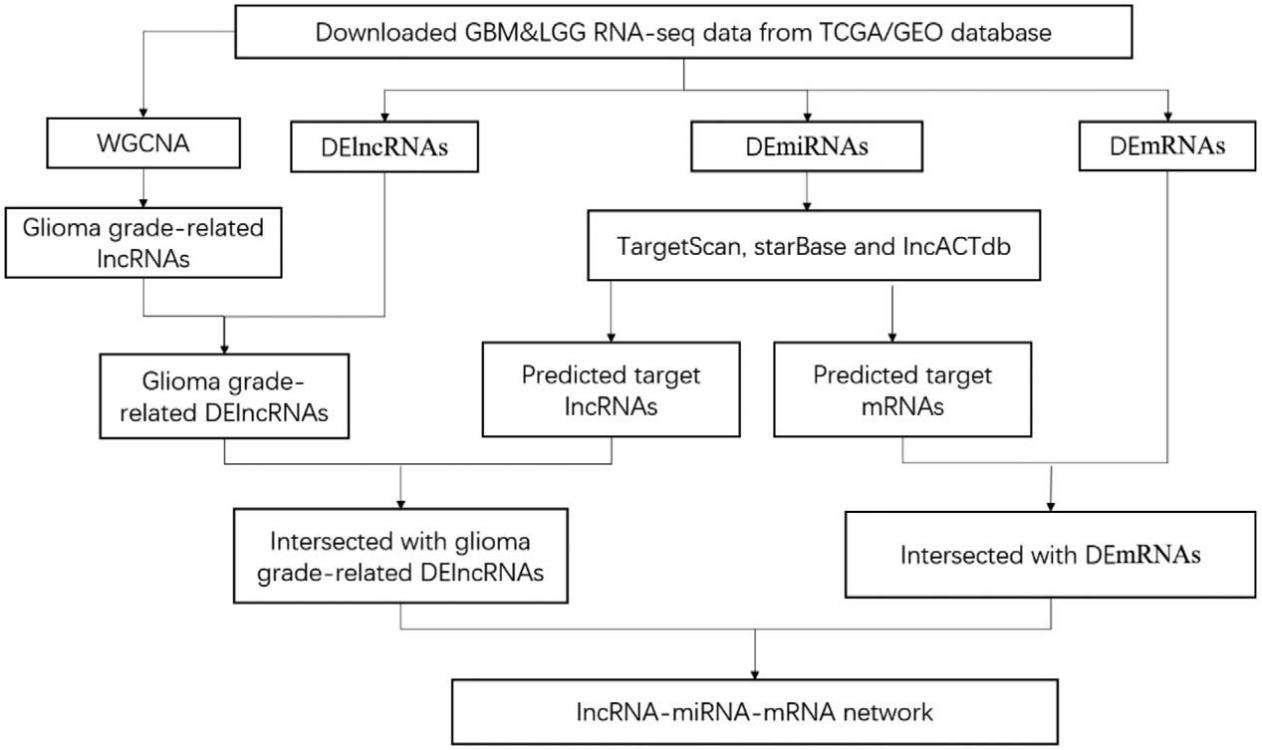
Flowchart of the construction of a ceRNA network. ceRNA = competing endogenous RNA, DElncRNAs = differentially expressed long non-coding RNAs, DEmiRNAs = differentially expressed microRNAs, DEmRNAs = differentially expressed mRNAs, GBM = glioblastoma, LGG = low-grade glioma.

According to the theory that lncRNAs can affect miRNAs and can act as miRNA sponges to further regulate mRNAs, we constructed a ceRNA network. The starBase (http://starbase.sysu.edu.cn/index.php) was used to predict the lncRNA/miRNA interactions based on DEmiRNAs.^[18]^ We predicted miRNA-targeted mRNA using TargetScan (http://www.targetscan.org/) and lncACTdb (http://www.bio-bigdata.net/LncACTdb/).^[19,20]^ We retained the intersection with the DEmRNAs and glioma grade-related DElncRNAs. Cytoscape version 3.7.2 was used to construct the lncRNA–miRNA–mRNA ceRNA network. The flowchart of ceRNA network construction is presented in Fig. 1.

### 2.5 Functional enrichment analysis and survival analysis

For further study of the functions of the mRNAs in the ceRNA network, the 57 upregulated mRNAs were selected to functional enrichment analysis with databases for annotation, visualization, and integrated discovery bioinformatics resources performed the gene ontology (GO) terms, Kyoto Encyclopedia of Genes and Genomes (KEGG) pathway, and Reactome pathway analyses. *P*-values of ≤.05 indicated enriched gene sets.

Hub mRNAs were selected according to the results of pathway analysis; for the purposes of this study, the lncRNAs that shared miRNAs with hub mRNAs in the ceRNA network were referred to as hub lncRNAs. By combining the clinical data of GBM patients in TCGA, the association between hub lncRNAs and survival time was analyzed using the survival R package (Version: 2.43-3), multivariate Cox proportional hazards regression mode was established based on the age, sex, and corresponding gene expression of patients. The surv_cutpoint function in survival R package is used to select the optimal cutoff value for grouping. *P* < .05 was set as the cutoff value.

## 3 Results

### 3.1 Differentially expressed genes

**Figure 2:**
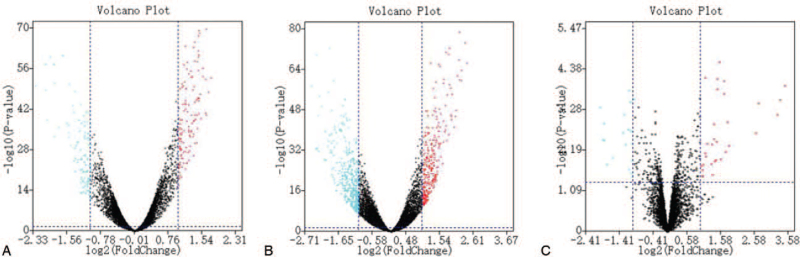
Volcano plots of the DEmRNAs (A), DElncRNAs (B), and DEmiRNAs (C). Log2 fold-change (cutoff = ±1, vertical lines) was plotted against the −log10 *P*-value (cutoff = 1.3, horizontal line). DElncRNAs = differentially expressed long non-coding RNAs, DEmiRNAs = differentially expressed microRNAs, DEmRNAs = differentially expressed mRNAs.

Using a cutoff threshold of |log2 FC| > 1 and an adjusted *P*-value of <.05 for the 156 GBM tissue samples compared with the 529 LGG tissue samples, we identified 666 DElncRNAs and 271 DEmRNAs (see Table S1, Supplemental Digital Content, which illustrates the results of differential gene expression). A total of 52 DEmiRNAs were identified from 10 GBM tissue samples in comparison with 15 LGG tissue samples (Table S1, Supplemental Digital Content). In total, 302 lncRNAs, 164 mRNAs, and 20 miRNAs were upregulated, while 364 lncRNAs, 107 mRNAs, and 32 miRNAs were downregulated. The volcano plots of these genes are shown in Fig. 2.

### 3.2 Identification of the gene coexpression modules

**Figure 3:**
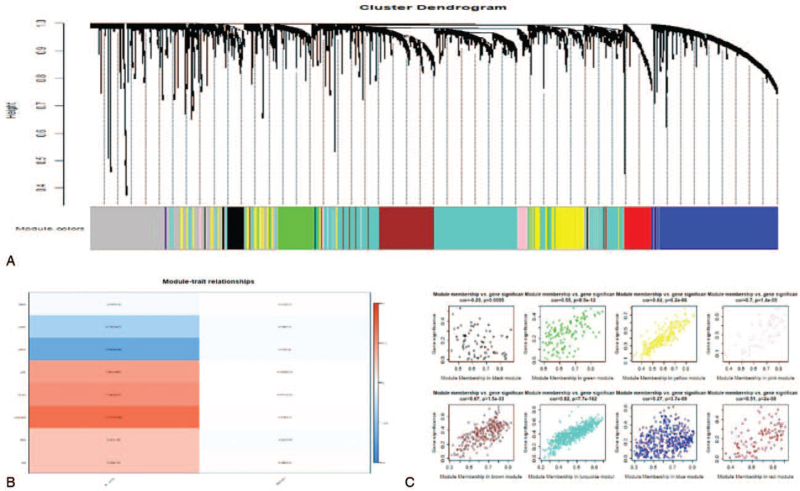
Clustering dendrograms and modules identified by WGCNA. (A) The clustering diagram and 9 modules for the lncRNA dataset. (B) The relationship between coexpression modules and glioma grade. The previous digit shows the module coefficient and the numbers in brackets represent *P*-values. (C) Correlations between genes and glioma grade in the modules. lncRNAs = long noncoding RNAs, WGCNA = weighted gene coexpression network analysis.

WGCNA is a systems biology method for describing correlation patterns among genes across microarray samples and exploring the hidden and biological patterns. Figure 3A shows hierarchical clustering of these 9711 mRNAs and the corresponding gene coexpression modules. The color bars correspond to 9 gene coexpression modules, including black, blue, brown, green, grey, pink, red, yellow, and turquoise modules; the gene numbers of these modules are 79, 462, 247, 132, 500, 57, 107, 243, and 661, respectively. Then, we identified coexpression modules associated with glioma grade. Figure 3B reveals the module significance. The yellow module (cor = −0.69, *P* = 9 × 10^−98^) and the turquoise module (cor = 0.71, *P* = 7 × 10^−106^) are highly correlated with glioma grade. We further analyzed the correlation between genes in modules and glioma grade, revealing that the yellow module (cor = 0.84, *P* = 6.2 × 10^−66^) and the turquoise module (cor = 0.82, *P* = 7.7 × 10^−162^) have higher correlations (Fig. 3C). Finally, we obtained 904 glioma grade-related lncRNAs from the turquoise module and the yellow module (see Table S2, Supplemental Digital Content, which illustrates the lncRNAs of the turquoise module and the yellow module), as well as 225 glioma grade-related DElncRNAs from the intersection of glioma grade-related lncRNAs and DElncRNAs.

### 3.3 Construction of a ceRNA network

**Figure 4:**
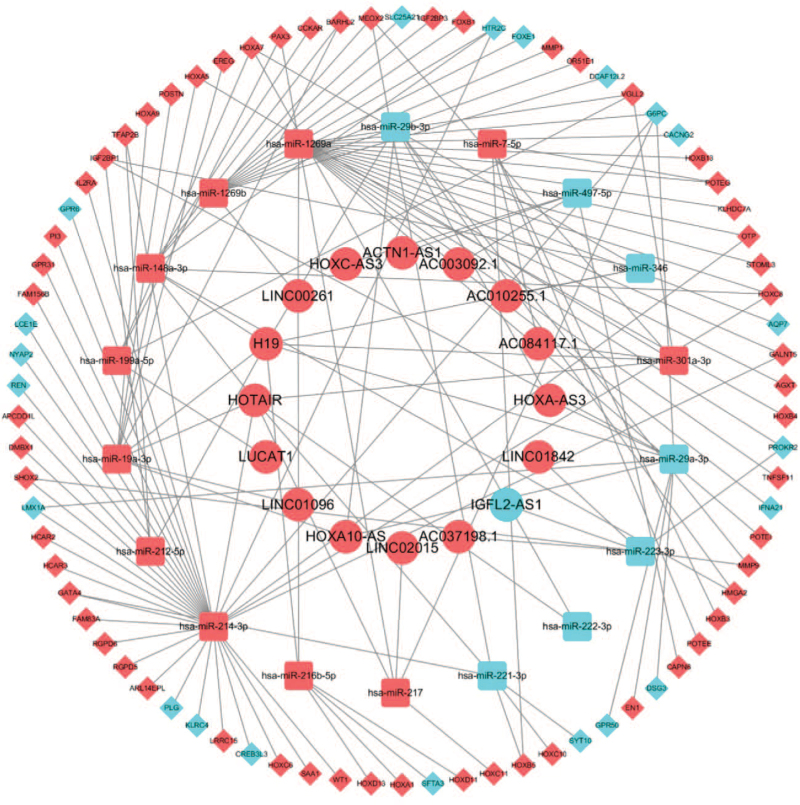
The lncRNA–miRNA–mRNA ceRNA network. Red balls, upregulated lncRNAs; blue balls, downregulated lncRNAs; red squares, upregulated miRNAs; blue squares, downregulated miRNAs; red diamonds, upregulated mRNAs; blue diamonds, downregulated mRNAs. ceRNA = competing endogenous RNA, lncRNAs = long noncoding RNAs, miRNA = microRNA, mRNA = messenger RNA.

To better understand the effects of lncRNAs on mRNAs mediated by the combination with miRNAs in the malignant differentiation of LGG to GBM, we constructed a ceRNA network based on the abovementioned data and used Cytoscape version 3.7.2 to visualize the network. Eighteen DElncRNAs interacted with 25 DEmiRNAs retrieved from the miRcode database (see Table S3, Supplemental Digital Content, which illustrates the 18 DElncRNAs interacted with 25 DEmiRNAs). Then, we searched for DEmRNAs based on 52 DEmiRNAs in the lncACTdb and TargetScan databases. Ultimately, 172 DEmRNAs could interact with 42 of the 52 DEmiRNAs according to these 2 databases (see Table S4, Supplemental Digital Content, which illustrates the 42 DEmiRNAs interacted with 172 DEmRNAs). According to information provided in Tables S3, Supplemental Digital Content and S4, 16 DElncRNAs, 18 DEmiRNAs, and 78 DEmRNAs were used to establish a ceRNA network (Fig. 4).

### 3.4 Upregulated DEmRNA functional enrichment analysis and survival analysis for the hub lncRNAs

**Figure 5:**
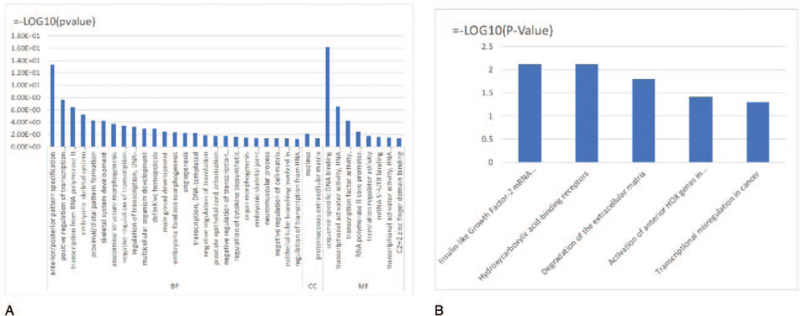
Functional analysis of upregulated mRNAs in the ceRNA network. (A) GO terms. (B) KEGG and Reactome pathway enrichment terms. (C) Ten hub mRNAs are enriched in 4 pathways. BP = biological function, CC = cellular component, ceRNA = competing endogenous RNA, FC = foldchange, GO = gene ontology, KEGG = Kyoto Encyclopedia of Genes and Genomes, MF = molecular function, mRNA = messenger RNA.

Functional enrichment analysis based on 57 upregulated DEmRNAs in ceRNA networks provided 35 GO terms, including 26 terms in the biological function category, 2 terms in the cellular component category, and 8 terms in the molecular function category (Fig. 5A) (see Table S5, Supplemental Digital Content, which illustrates the results of enrichment analysis). The top 10 GO terms were sequence-specific DNA binding, anterior/posterior pattern specification, positive regulation of transcription from RNA polymerase II promoter, transcriptional activator activity, transcription from RNA polymerase II promoter, embryonic skeletal system morphogenesis, proximal/distal pattern formation, transcription factor activity, skeletal system development, and anatomical structure morphogenesis.

KEGG and Reactome pathway analysis revealed that the upregulated DEmRNAs are significantly enriched in 5 pathways (Fig. 5B) (see Table S6, Supplemental Digital Content, which illustrates the results of pathway analysis). Four pathways—insulin-like growth factor-2 mRNA-binding proteins, degradation of the extracellular matrix, activation of anterior homeobox (HOX) genes in hindbrain development during early embryogenesis, and transcriptional misregulation in cancer—are closely related to cancer pathogenesis, progression, and malignant differentiation.^[21–23]^ Ten hub mRNAs (IGF2BP1, IGF2BP3, CAPN6, MMP9, MMP1, HOXB3, HOXB4, HOXA1, HMGA2, and WT1) are enriched in these 4 pathways (Fig. 5C).

**Figure 6:**
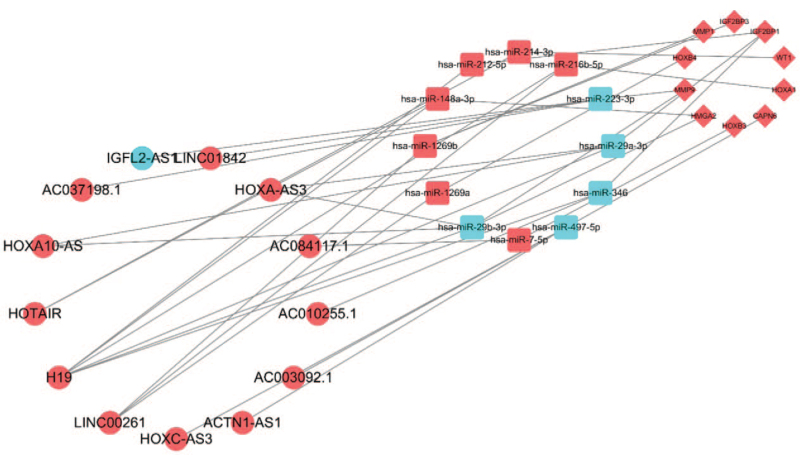
The ceRNA network of hub RNAs. Red balls, upregulated hub lncRNAs; blue balls, downregulated hub lncRNA; red squares, upregulated miRNAs; blue squares, downregulated miRNAs; red diamonds, upregulated hub mRNAs. Sankey diagram, each rectangle represents a gene, and the connection degree of each gene is visualized based on the size of the rectangle. ceRNA = competitive endogenous RNA, lncRNAs = long noncoding RNAs, miRNA = microRNA.

**Figure 7:**
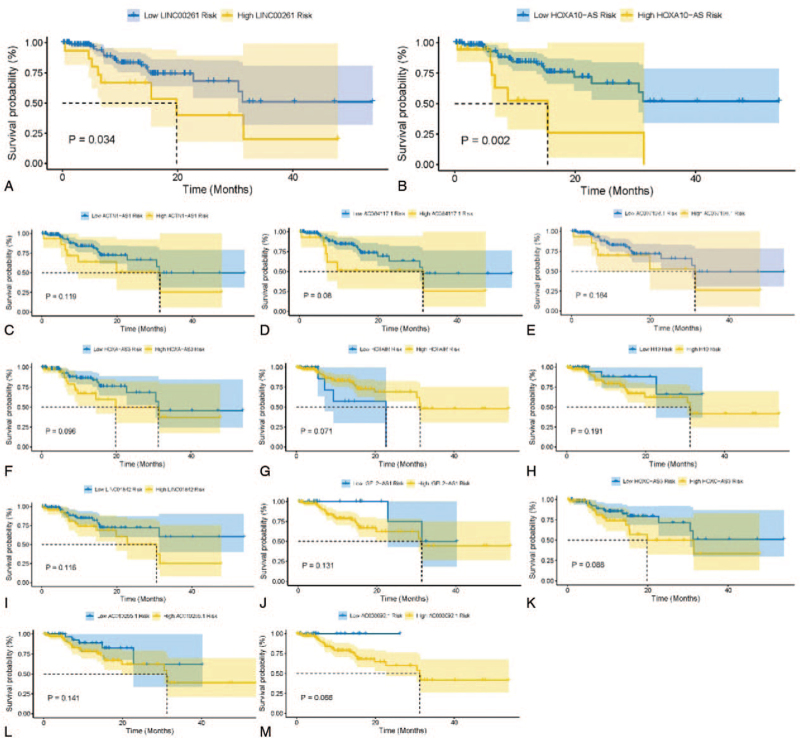
Kaplan–Meier survival curves of 9 hub lncRNAs associated with overall survival in patients with GBM. GBM = glioblastoma, lncRNAs = long noncoding RNAs.

We selected 13 hub lncRNAs that share miRNAs with hub mRNAs in the ceRNA network for survival analysis (Fig. 6). Among the 13 hub lncRNAs, the overall survival was negatively correlated with 2 hub lncRNA transcripts (LINC00261 and HOXA10-AS) (*P* < .05; Fig. 7A–M).

## 4 Discussion

The ceRNA concept has been proposed recently. It describes a class of RNAs with miRNA binding sites and can thus compete with the miRNA-targeted mRNAs for the miRNAs. Understanding of ceRNA crosstalk has shown that miRNAs and their targets establish complex ceRNA networks.^[24]^ Multiple studies have suggested that abnormal lncRNAs play key roles in GBM development.^[25–27]^ However, few studies on ceRNAs have focused on the transformation of LGG to GBM. Additionally, rare yet reliable lncRNAs, miRNAs, and mRNAs related to GBM can be treated as molecular biomarkers for detecting GBM and stratifying the GBM risk.

In this work, we analyzed differentially expressed lncRNAs, mRNAs, and miRNAs with the GBM tissue samples relative to the LGG tissue samples. WGCNA was used to identify glioma grade-related DElncRNAs. Based on the comprehensive integration of the data for DEmiRNAs, DEmRNAs, and grade-related DElncRNAs, we constructed the lncRNA–miRNA–mRNA ceRNA network.

In the ceRNA network, G6PC, HOXC8, VGLL2, HTR2C, MEOX2, HOXA7, and IGF2BP1 have the highest connection degree (connection degree ≥3) (see Table S7, Supplemental Digital Content, which illustrates the information of the ceRNA network), Therefore, we concluded that they might exert a strong influence on the progression of LGG to GBM. HOXC8, MEOX2, and HOXA7 belong to the HOX family of genes. Preliminary studies have indicated that there is an induction of HOX gene expression in differentiating LA-N-5 neuroblastoma cells.^[28]^ IGF2BP1, the most studied IGF2BP1 family member, regulates localization, translation, turnover, and expression of key oncogenes, including glioma-associated oncogene homolog 1, insulin-like growth factor 2, c-Myc, and several others.^[21,29,30]^ One study showed that aG6PC-deficient mice have a high risk of developing hepatocellular adenomas.^[31]^ VGLL2 encodes a protein that may act as a cofactor of TEF-1-regulated gene expression during skeletal, VGLL2-related fusions, and play an important role in rhabdomyosarcoma.^[32–34]^ HTR2C encodes a 7-transmembrane G-protein-coupled receptor closely correlated with epilepsy, depression, and mental disorders. There is no obvious research report available related to glioma focusing on G6PC, VGLL2, and HTR2C, from which 2 inferences can possibly be drawn: first, these are not necessarily related to glioma or, second, whether these key targets are related to glioma has not been studied yet, which provides a theoretical reference for further evaluation.

In addition to mRNAs, miRNAs should receive extensive attention. Undoubtedly, research related to tumorigenicity in terms of miRNA regulation is critically needed. miRNAs are RNA molecules of approximately 22 nucleotides in length, which bind to their respective target genes and exert their influence on gene expression by either inhibiting protein translation or degrading mRNA.^[35]^ We noted that the DEmiRNA hsa-miR-214-3p (connection degree = 33) and hsa-miR-1269a (connection degree = 18) have the highest connection degrees in the ceRNA network, suggesting an obvious influence of hsa-miR-214-3p and hsa-miR-1269a on GBM malignant differentiation and prognosis (Table S7, Supplemental Digital Content). Wang et al^[36]^ reported that, through the sponging effect to induce hsa-miR-214-3p downregulation, the upregulation of lncRNA XIST may suppress epithelial ovarian cancer development. Liu et al^[37]^ revealed that lncRNA HCP5 is highly expressed in pancreatic cancer and the development of gemcitabine-resistant pancreatic cancer cells encompassing the processes of proliferation, invasive, migration, cell apoptosis, and autophagy through the miR-214-3p–HDGF axis. Kitdumrongthum et al^[38]^ found that hsa-miR-214-3p was down-regulated by 1000 to 2000-fold in cholangiocarcinoma exosomes. A pan-cancer analysis showed that compared with non-tumorous samples, hsa-miR-1269a was significantly upregulated and frequently found (at least 60%) in tumor samples of 8 cancers, including GBM.^[39]^ The correlations between these 2 miRNAs and GBM transformation and prognosis require further experimental exploration.

The 57 upregulated DEmRNAs in the ceRNA network appeared to be significantly enriched in 5 pathways. Four pathways—insulin-like growth factor-2 mRNA-binding proteins, degradation of the extracellular matrix, activation of anterior HOX genes in hindbrain development during early embryogenesis, and transcriptional misregulation in cancer—are closely related with cancer pathogenesis, progression, and transformation. Insulin-like growth Factor-2 mRNA binding proteins modulate cell polarization, adhesion, and migration in tumor-derived cells; moreover, they are highly associated with cancer metastasis and the expression of oncogenic factors (KRAS, MYC, and MDR1).^[21]^ A correlation has been demonstrated between the level of extracellular matrix molecule expression and the invasive and metastatic aggressiveness of tumor cells.^[22]^ One study reported that HOXB1, HOXB2, and HOXB3 are able to positively interact with the Otx2 upstream regulatory sequence in an embryonal carcinoma cell line.^[40]^

Regarding the 10 hub mRNAs enriched in these 4 pathways, there are 13 hub lncRNAs that share miRNAs with these 10 hub mRNAs in the ceRNA network. Among the 13 hub lncRNAs, the overall survival outcome was significantly related to 2 hub lncRNA transcripts (LINC00261 and HOXA10-AS). Among these 13 hub lncRNAs, H19, HOTAIR, LINC00261, and HOXA10-AS had the highest connection degrees in the ceRNA network (connection degree ≥ 3) (Table S7, Supplemental Digital Content); therefore, we inferred that they might be the key lncRNAs in the malignant differentiation of glioma. The imprinted oncofetal lncRNA H19 is expressed in the embryo, downregulated at birth, and then reappears in tumors, with accumulating data supporting that H19 is one of the major genes in cancer. H19 upregulation allows cells to enter a “selfish” survival mode in response to stress conditions, such as the destabilization of the genome and hypoxia, by accelerating their proliferation rate and increasing the overall cellular resistance to stress. This response is tightly correlated with the nullification, dysfunction, or significant downregulation of the master tumor suppressor gene P53.^[41]^ A study suggested a role for H19 in contributing to GBM malignancy differentiation and the maintenance of its stem cell properties.^[42]^ HOTAIR has been involved in the evolution of several primary tumors, wherein the increase in HOTAIR expression endorses invasion and metastasis.^[43]^ Pastori et al^[44]^ demonstrated that HOTAIR is overexpressed in GBM and controls GBM cell proliferation. Meanwhile, the knockdown of HOTAIR reduced proliferation and increased the apoptosis of GBM cells in vitro and in vivo. Importantly, HOTAIR is part of the proliferative pathway controlled by bromodomain reader proteins, which are therapeutic targets in GBM and other cancers. LINC00261 is an epigenetically regulated tumor suppressor essential for the activation of the DNA damage response,^[45]^ Several studies have proved that LINC00261 concerned to kinds of cancer cell proliferation, migration, and invasion.^[46–48]^ A previous study discovered that HOXA10-AS was significantly upregulated in glioma tissues and cell lines; along these lines, increased HOXA10-AS expression levels were associated with higher grades of glioma. The knockdown of HOXA10-AS inhibited glioma cell proliferation and increased cell apoptosis rates.^[49]^ HOXA10-AS was identified as a risk factor for oral squamous cell carcinoma and its expression was positively associated with tumor grade.^[50]^ A previous study discovered that epithelial ovarian cancer HOXA11-AS has a tumor suppressor function in EOC which may be enhanced by the T allele.^[51]^

Of note, this study had certain limitations. In particular, there was a lack of experimental validation in vitro and in vivo. The present results and conclusions may serve as a foundation for the establishment of mechanistic hypotheses as a basis for further experiments on clinical samples and cell lines.

In conclusion, a ceRNA network, including 16 DElncRNAs, 18 DEmiRNAs, and 78 DEmRNAs, was successfully constructed. Importantly, 2 lncRNAs (LINC00261 and HOXA10-AS) were remarkably correlated with the prognosis of patients with GBM in TCGA. Our research provides novel insights that will increase the understanding of the malignant differentiation-related ceRNA networks in glioma. Furthermore, the candidate lncRNAs, miRNAs, and mRNAs involved in our ceRNA network can be further evaluated as potential therapeutic targets and prognostic biomarkers for GBM.

## Acknowledgments

The authors thank LetPub (www.letpub.com) for its linguistic assistance during the preparation of this manuscript.

## Author contributions

Xin Li conceived and designed the study, and drafted the manuscript. Jingwen Zhang and Min Zhang acquired, analyzed, and interpreted the data, and conducted the statistical analysis. Xin Li and Jingwen Zhang acquired and interpreted data, and revised the manuscript for important intellectual content. All authors read and approved the final manuscript.

**Conceptualization:** Xin Li.

**Data curation:** Xin Li, Jingwen Zhang, Min Zhang.

**Funding acquisition:** Xianghua Qi.

**Methodology:** Jing Teng.

**Supervision:** Shiyuan Wang, Jing Teng.

**Validation:** Xin Li, Jingwen Zhang, Min Zhang, Xianghua Qi, Shiyuan Wang, Jing Teng.

**Visualization:** Xin Li, Min Zhang.

**Writing – original draft:** Xin Li, Min Zhang, Jing Teng.

## Supplementary Material

Supplemental Digital Content

## Supplementary Material

Supplemental Digital Content

## Supplementary Material

Supplemental Digital Content

## Supplementary Material

Supplemental Digital Content

## Supplementary Material

Supplemental Digital Content

## Supplementary Material

Supplemental Digital Content

## Supplementary Material

Supplemental Digital Content
